# Resistance to Radiotherapy in Cancer

**DOI:** 10.3390/diseases13010022

**Published:** 2025-01-17

**Authors:** Almaz A. Akhunzianov, Elvira V. Rozhina, Yuliya V. Filina, Albert A. Rizvanov, Regina R. Miftakhova

**Affiliations:** 1 Institute of Fundamental Medicine and Biology, Kazan Federal University, 420008 Kazan, Russia; 2 Division of Medical and Biological Sciences, Tatarstan Academy of Sciences, 420111 Kazan, Russia

**Keywords:** cancer, radiotherapy, radioresistance, biomarkers, cellular metabolism, mitochondria

## Abstract

Radiation therapy or radiotherapy is a medical treatment that uses high doses of ionizing radiation to eliminate cancer cells and shrink tumors. It works by targeting the DNA within the tumor cells restricting their proliferation. Radiotherapy has been used for treating cancer for more than 100 years. Along with surgery and chemotherapy, it is one of the three main and most common approaches used in cancer therapy. Nowadays, radiotherapy has become a standard treatment option for a wide range of cancers around the world, including lung, breast, cervical, and colorectal cancers. Around 50% of all patients will require radiotherapy, 60% of whom are treated with curative intent. Moreover, it is commonly used for palliative treatment. Radiotherapy provides 5-year local control and overall survival benefit in 10.4% and 2.4% of all cancer patients, respectively. The highest local control benefit is reported for cervical (33%), head and neck (32%), and prostate (26%) cancers. But no benefit is observed in pancreas, ovary, liver, kidney, and colon cancers. Such relatively low efficiency is related to the development of radiation resistance, which results in cancer recurrence, metastatic dissemination, and poor prognosis. The identification of radioresistance biomarkers allows for improving the treatment outcome. These biomarkers mainly include proteins involved in metabolism and cell signaling pathways.

## 1. Introduction

According to statistics, cancer is one the most prevalent causes of death around the world; there were around 20 million new cases and 9.7 million cancer-related deaths worldwide in 2022 [1]. Radiation therapy or radiotherapy is a treatment approach based on utilizing high doses of ionizing radiation to target the DNA within the tumor cells. Radiation therapy has been used for cancer treatment for more than 120 years. Over this period, it has become one of the main types of cancer therapy. Currently, it represents a cost-effective and important treatment option for patients. It is a standard treatment option for a wide range of tumors, including some of the most common cancers: lung, breast, cervical, and colorectal cancers. Radiotherapy can be used (1) as a primary treatment for cancer; (2) after surgery (adjuvant therapy) to eliminate any remaining cancer cells; (3) before surgery (neoadjuvant therapy) to shrink tumors; and (4) to relieve symptoms caused by cancer (palliative care). According to the International Agency for Research on Cancer, nearly 50% of patients will require radiotherapy, 60% of whom are treated with curative intent [2]. Only 35% of radiotherapy units (4400) are located in developing countries with 85% of the world population; therefore, less than 20% of cancer patients in these countries have access to radiotherapy units. Around 48% of all cancer patients have indications for either curative (34%) or palliative (14%) radiotherapy. It provides 5-year overall survival benefit in 2.4% of patients [3]. It is frequently used to treat the most common cancer types, such as lung, breast, cervical, and colorectal cancers. In Europe, the tumors that make up the highest number of patients in a radiotherapy departments in are breast (396,891), lung (315,197), prostate (243,669), head and neck (108,194), and rectal cancers (99,493) [4]. On average, these numbers are expected to increase by 16% by the end of 2025. Therefore, in this review, we aim to discuss the molecular mechanisms associated with radioresistance. We have also summarized radiotherapy resistance biomarkers and potential targets that may be used to improve treatment outcomes, reduce the cancer burden, and enhance the quality of life of cancer patients. But first of all, we wish to discuss the history, types, and recent advances in radiation therapy.

## 2. Materials and Methods

We performed a strategic search through PubMed to find studies regarding radiotherapy and radioresistance biomarkers. The keywords used for the search were “radiotherapy history”, “types of radiotherapy”, “radiotherapeutic resistance”, and “radiotherapeutic resistance and metabolism”. In total, we found 3364 manuscripts. We selected articles published in English up to June 2024 based on the publication date and relevance to the topic. After excluding duplicates, we analyzed the abstract and text and selected 100 valuable articles. Original figures were created using BioRender.

## 3. History and Types of Radiotherapy

Shortly after the discovery of X-rays by German physicist Wilhelm Conrad Roentgen, Nikola Tesla, the famous Serbian–American inventor, wondered if it might be possible to “load” this new type of ray with cancer-fighting chemicals and project them into the body to cure cancer. Emil Grubbe was the first American physician who used X-rays for cancer treatment in 1896. The first successful use of X-rays in the treatment of cancer was reported by Swedish radiology pioneer Thor Stenbeck in 1898 [5]. One year later, a positive effect of exposure to X-rays in skin cancer therapy was confirmed by Tage Sjögren. However, it is important to note that T. Stenbeck and T. Sjögren combined this initial treatment with surgery. In the beginning of the 20th century, many scientists reported the promising results of using this new type of cancer treatment. Further improvements and specialization of treatment tubes allowed for aiming X-rays at cancer tumors of the esophagus, larynx, and cervix uteri.

Meanwhile, radium-caused skin inflammation was reported. French dermatologist Ernest Besnier suggested that it could be used for the same purposes as X-rays. Soon after, attempts to use radium for therapy began. It was clear that the application of radium resulted in tumor shrinking [6]. Over time, physicians came to a conclusion that X-rays and radium therapies have different advantages. The use of radium was preferred for a localized treatment. Thus, these cases can be considered as the origin of brachytherapy, which has been commonly used for the treatment of certain types of cancers (breast, prostate, skin) to this day.

At the same time, concerns were brought up by scientists almost immediately after these new types of therapy had become popular. Emil Grubbe reported painful lesions after radioactive exposure [7]. Physicians were forced to conclude that X-rays and radium could not only cure, but also harm and even kill. By 1910, the primary enthusiasm about the miraculous features of this new type of therapy had dimmed. There were lots of reports about negative physiological effects, from scars and depilation to fatal leukemia and anemia. Physicians found out that radiation had the effect of worsening some cancers. At the International Congress of Oncology, Henri Coutard reported that laryngeal cancer could be cured without serious negative effects on patients [8]. He laid the foundation for modern radiotherapy in 1934. He was the first radiologist who assumed that both cancer and normal cells had the same sensitivity to radioactive exposure; physicians had to use this new method of treatment carefully with minimal negative physiological side effects. He designed the fractionated dosage approach, and from 1935, physicians began to follow this cancer treatment plan [9]. This approach has not lost its relevance, even to this very day.

Advances in technology have fostered the development of more effective and sophisticated treatment and radiation delivery techniques. Nowadays, conformal radiation therapy (CRT) is the most commonly used type of external beam radiation therapy. CRT allows for aiming beams from several directions and sparing nearby structures. Thus, higher doses of radiation can be targeted to the cancer tumor. In conformal radiotherapy, a linear accelerator is combined with a multi-leaf collimator. It consists of small metal bars of a material with a high atomic number (usually, tungsten is used) that can move independently to block the radiation beams and conform them to the shape of the tumor. Brief information about different types of conformal radiation therapies (IGRT, IMRT, PRT) is presented below.

As mentioned above, the main purpose of radiotherapy is to affect tumors and at the same time avoid surrounding healthy tissues and organs. Modern techniques allow for locating tumors more precisely for accurate delivery of treatment; such inspection is carried out in Image Guided Radiotherapy (IGRT). Images and virtual models of tumors and their exact location can be acquired before (2D and 3D) and even during the session (4D) of radiotherapy by means of computed tomography, magnetic resonance imaging, ultrasound, and X-ray imaging [10]. In 4D-IGRT, infractional (during one session of therapy) motions of targeted regions are taken into account. Cone Beam Computed Tomography (CBCT) is one of the most popular IGRT systems for external beam radiotherapy. It provides high-resolution images of soft tissues. In CBCT, divergent X-rays form a cone [11]. The place of interest is located at the center of the cone beam field. During 200-degree single rotation, an initial data set is acquired and sent to scanning software, which reconstructs a three-dimensional virtual model.

Intensity Modulated Radiotherapy (IMRT) is a type of radiotherapy that adjusts the radiation beams to the shape of the tumor, and the intensity profile of the radiation beams can be modulated. This type of radiotherapy was introduced in the early 1990s as a further advancement of conformal radiotherapy. Advanced treatment planning algorithms optimize the position of the multi-leaf collimator’s bar. In IMRT, beams are divided into several small beamlets with different intensity. This allows for the delivery of different doses of radiation across the tumor [12]. Additionally, during IMRT, a concave area at the edge of the radiation can be created. This allows for avoiding high doses of radiation to the structures like the spinal cord. Thus, the risk of long-term side effects is reduced.

Although photon beams are the most commonly used type of external radiation in cancer therapy, proton therapy confers a dosimetric advantage [13]. In conformal proton radiation therapy, cancer tumors are irradiated by proton beams. The proton beam has low incident energy. It does not broaden, and the dose is mainly delivered over the last few millimeters of the protons’ range (the Bragg peak). Protons almost do not penetrate into surrounding tissue further beyond that distance. As a result, the energy of accelerated particles is accumulated in the targeted region within the tumor. Carbon-12 (12C) atoms are also widely used in proton beam radiotherapy. The disadvantage of proton beam radiotherapy is the very high cost of proton cyclotron; also, there is still significant uncertainty around the exact proton beam field for an individual patient.

As mentioned above, during brachytherapy, the radioactive source is placed directly inside or near the tumor. This allows for delivering larger doses of radiation to cancer tissues than in external beam radiotherapy. Because of the intratumoral location of the radioactive source, less radiation goes to nearby tissues. Consequently, the rate of negative physiological side effects is reduced too [14]. The gold seeds with radon or cobalt were commonly used until the 60s of the 20th century. Later, these materials were replaced by radioactive tantalum and gold. Nowadays, iridium-192 is the most commonly used source in brachytherapy. Nevertheless, different materials with different half-lives may be chosen for treatment depending on the type of cancer tumor. The radioactive source is implanted either permanently (for the rest of the patient’s life), or temporarily. Only low-dose-rate brachytherapy (LDR-BT) may be given with permanent implants [15]. The permanent source usually loses its radioactivity after several weeks [16]. Temporary implants allow for the administration of both LDR-BT and high-dose-rate brachytherapy (HDR-BT). During HDR-BT, thin catheters are placed nearby or in the tumor. Later, catheters are attached to a remote afterloading system (RALS). The radioactive source is “afterloaded” or pushed into catheters by RALS [17]. The dwell time and dwell positions of sources can be controlled remotely. After treatment, the radioactive seeds are withdrawn, and the catheters are removed too.

Stereotactic radiosurgery (SRS) is high-dose-rate radiotherapy commonly used for the treatment of brain cancer tumors or tumors located in the head [18]. SRS and similar stereotactic body radiotherapy (SABR) are used for the treatment of small tumors with a clear margin, as well as secondary or inoperable tumors. In the case of radiosurgery, no actual surgical cuts are made, and a single high-dose fraction is precisely delivered to a well-defined tumor [19]. The location and shape of the tumor are preliminarily examined, like in IMRT, by CT or MR scans. For accurate targeting during a session, stereotactic masks and head frames are used. These devices immobilize the patient’s head during the session. In contrast to SRS in stereotactic radiotherapy, the treatment is divided into sessions, and multiple fractions of radiation are given. An advanced CyberKnife radiosurgery system, invented by John Adler, Peter, and Russel Schonberg, allows for treating small benign and malignant tumors in several sessions without using the stereotactic frame [20]. The person’s position is tracked by X-ray cameras. A robotic system adjusts the linear accelerator and the radiation beams’ direction to the patient’s slight movements.

FLASH radiotherapy is an innovative approach to cancer treatment that delivers ultra-high doses of radiation in a fraction of a second, significantly faster than traditional radiotherapy [21]. This technique, which uses ultra-high dose rates exceeding 40 Gy per second, is designed to target tumors while sparing surrounding healthy tissues [22]. The underlying principle is known as the “FLASH effect”, where normal tissues exhibit less damage when exposed to such rapid doses compared to conventional radiotherapy methods. FLASH radiotherapy is based on the differences in cellular response to high-dose radiation between tumor cells and normal cells [23].

The mechanism behind the “FLASH effect” is not fully understood but is suggested to involve differential oxygen consumption in tissues. This effect involves the rapid depletion of local oxygen in cells during ultra-high-dose radiation [24]. The quick oxygen depletion induces local hypoxia, influencing the various responses observed between surrounding healthy cells and tumor cells. Tumor cells, which often exist in a hypoxic environment, are less able to recover from the high-energy radiation damage. The therapeutic effect also involves the generation of reactive oxygen species (ROS) and hydrogen peroxide products during exposure to radiation [25]. Compared to tumor cells, healthy cells more efficiently eliminate these dangerous substances due to higher catalase activity. Additionally, the rapid treatment delivery of flash radiotherapy reduces side effects and treatment times, which can improve overall patient experience.

Despite its promise, flash radiotherapy has not achieved widespread clinical adoption yet; it is still in its experimental stages. Technical barriers include the development of equipment capable of delivering such precise and high-speed doses and ensuring consistent results across different tumor types. Nonetheless, flash radiotherapy could revolutionize cancer radiotherapy, making treatments not only faster but also safer and more effective. Clinical trials are ongoing to establish safety, efficacy, and long-term outcomes in patients. The first clinical study of FLASH radiotherapy was conducted in 2018. A 75-year-old patient diagnosed with T-cell skin lymphoma received a total dose of 15 Gy delivered in 10 pulses, each pulse lasting for 1 microsecond. The tumor response was rapid, complete, and durable with a follow-up time of 5 months [26]. Only grade 1 dermatitis and edema were observed after this initial treatment. Thus, the first treatment was feasible and relatively safe for this patient. In 2021, 10 patients with painful bone metastases were given 8 Gy of radiation in a single fraction, delivered at ≥40 Gy per second (NCT04592887). Pain, use of pain medications, and adverse events were measured; the median follow-up time was 4.8 (2.3–13.0) months. Seven patients experienced complete or partial pain relief. In 6 of the 12 sites (50%), patients reported a complete response [27]. Side effects from treatment were mild and consistent with conventional radiotherapy. In recent years, clinical trials of this innovative therapy have progressed to Phase II [28]. Hence, flash radiotherapy offers a way to minimize side effects while maintaining, or even improving, the efficacy of cancer treatment. Predictive parameters for FLASH radiotherapy are critical for optimizing its efficacy and safety. Tumor type and radiosensitivity play key roles in treatment response. Experiments on animal models demonstrated that some tissues, like the brain and lungs, show a more pronounced “FLASH effect” [29,30]. Larger volumes of exposed tissue may not experience uniform effects due to dose rate variations. Research continues to determine the optimal dose rate for specific tumors, aiming to maximize the therapeutic ratio while minimizing side effects for surrounding healthy tissues and organs.

## 4. Radiotherapy Resistance Biomarkers

**Figure 1:**
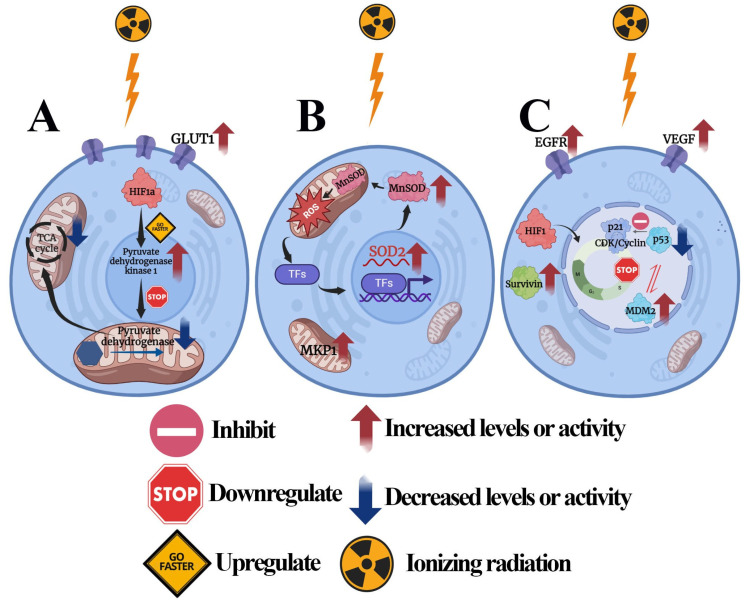
Molecular mechanisms of resistance to radiotherapy: **TCA**—tricarboxylic acid cycle; **HIF1a**—hypoxia-inducible factor 1-alpha; **GLUT1**—glucose transporter 1; **ROS**—reactive oxygen species; **SOD**—superoxide dismutase; **TFs**—transcription factors; **MKP1**—mitogen-activated protein kinase phosphatase-1; **EGFR**—epidermal growth factor receptor; **VEGF**—vascular endothelial growth factor; **CDK**—cyclin-dependent kinase; **MDM2**—mouse double minute 2 homolog. (**A**) Radiotherapy resistance and glycolysis: GLUT1 and PDK1 overexpression is associated with radiotherapy resistance. HIF1α upregulates pyruvate dehydrogenase kinase 1 (PDK1) expression. PDK1 phosphorylates and inactivates the PDH enzyme complex, which converts pyruvate to acetyl-coenzyme A, thus suppressing the tricarboxylic acid (TCA) cycle and decreasing the oxygen consumption rate. (**B**) Radiotherapy resistance and mitochondrial metabolism: MnSOD protects cells from ROS-induced damage and contributes to radiotherapy resistance. MKP1 limits the accumulation of phosphorylated JNK and, thus, prevents the induction of apoptosis. (**C**) Radiotherapy resistance and cell signaling pathways: MDM2 represses p53 transcriptional activity and contributes to radioresistance. P21 (a major target of p53) promotes cell cycle progression at the G1 phase in radioresistant cells. Survivin blocks the apoptotic pathway and enhances radiation resistance by inhibiting caspase 9. Figures were created in BioRender.

It has previously been estimated that 52% of newly diagnosed cancer patients will require radiation therapy [31]. Radiotherapy is widely used as definitive, neoadjuvant, or adjuvant treatment [32]. Many factors influence tumor response to irradiation: total received dose, fractionation, and the intrinsic radiosensitivity of tumor cells [33]. Ionizing radiation directly affects the DNA, resulting in gene mutation and chromosome breakage. As research advanced, it became evident that lipids and proteins are also critical targets of radiation [34]. It induces lipid peroxidation and influences the post-transcriptional and post-translational modifications of proteins, leading to cell damage. Indirect damage from ionizing radiation is associated with the generation of ROS, which can further affect cellular components and metabolism (Figure 1). This damage can result in mutations, cell death, or the activation of cellular repair mechanisms, which are critical factors in the effectiveness of radiotherapy [35]. Additionally, ionizing radiation can also affect other cellular structures, such as membranes and receptors. It can mimic the binding action of ligands to the epidermal growth factor receptor (EGFR) and has been demonstrated to induce receptor dimerization, followed by autophosphorylation [36].

Tumor cell radioresistance contributes to therapy failure, ultimately leading to cancer recurrence and metastasis [37]. Hence, it is important to identify biomarkers for radiosensitivity and radioresistance. In recent years, significant efforts have been made to identify predictive and prognostic biomarkers for different types of cancer. Prognostic markers provide information about disease outcomes. Predictive markers indicate the benefits of a specific therapy for a particular patient [38]. For example, if some specific marker associated with relapse is detected at an early stage, patients may benefit from an alternative therapy. Additionally, radiotherapy resistance biomarkers could serve as a therapeutic target [39]. Radioresistance is a critical problem in cancer therapy. Stratification of patients based on tumor radiosensitivity is crucial to maximize the efficacy of medical resources and to improve the therapy outcome. Molecular mechanisms underlying tumor cells radioresistance are yet to be elucidated, although the first steps in this area have already given some valuable results (summarized in Table 1). Further, we want to present a brief overview of different molecular markers of radiotherapeutic resistance. Here, we start from metabolic markers of radioresistance, and then discuss some hallmark genes involved in cell cycle control and apoptosis. Finally, we highlight several circulating biomarkers that can be used as non-invasive tools to predict radiosensitivity and radioresistance.

## 5. Radiotherapy Resistance and Glycolysis

Metabolic reprogramming is considered to be one of the hallmarks of cancer [50]. Cancer cells take up large amounts of glucose to utilize it in aerobic glycolysis and generate energy [51]. Active glycolysis confers a powerful growth advantage, which promotes the uncontrolled proliferation and invasion of cancer cells [52]. On the contrary, alterations in metabolism can also promote the development and progression of cancer; e.g., in animal model experiments, liver carcinogenesis is associated with metabolic disorders and obesity [53]. Multiple studies indicate that metabolic changes in tumor cells contribute to radioresistance. For example, AKT oncogene activation leads to a metabolic switch to aerobic glycolysis, which in turn causes acquired radioresistance in human liver and cervical cancer cell lines [54]. The transient elevation of glycolysis promotes the rejoining of radiation-induced DNA strand breaks in cancer cells by activating homologous recombination and non-homologous end joining pathways, thus protecting cancer cells from the cytogenetic damage induced by ionizing radiation [55].

Glucose transporter 1 (GLUT1) is an integral membrane protein that mediates glucose diffusion across the cell membrane [56]. GLUT1 overexpression is associated with resistance to radiotherapy and adverse prognosis in oral squamous cell carcinoma [41]. The possible explanation of the linkage between GLUT1 expression and radiation resistance can be found in cardiology [40]. Glucose-dependent inhibition of the mitochondrial apoptotic pathway protects cardiac myocytes against apoptosis. Another possible protective mechanism involves caspase-2 phosphorylation and inhibition by generated NADPH [57]. Patients with elevated expression of GLUT1 demonstrate greater resistance to radiotherapy and shorter progression-free survival in cervical squamous cell carcinoma [58]. GLUT1 is an important target in cancer therapy, and its inhibition may represent an effective approach to overcoming cancer cell resistance; the small molecule WZB117 inhibits GLUT1 expression and sensitizes breast cancer cells to ionizing radiation [49].

Pyruvate dehydrogenase kinase (PDK) is a kinase enzyme located in the mitochondrial matrix and involved in glucose metabolism [59]. It phosphorylates the pyruvate dehydrogenase (PDH) E1α subunit and inactivates the PDH enzyme complex that converts pyruvate to acetyl-coenzyme A, thus suppressing the tricarboxylic acid (TCA) cycle [44]. The expression of its inhibitor pyruvate dehydrogenase kinase (PDK) was shown to correlate with radioresistance in glioblastoma cells [60]. Immunohistochemical analysis demonstrated a positive correlation between PDK3 levels and the severity of colon cancer, as well as a negative correlation with disease-free survival among patients [61]. High glycolytic state is well known to correlate with radiotherapeutic resistance of tumor cells. After exposure to ionizing radiation, oxygen molecules can react with free radicals and stabilize the integrity and chemical composition of the DNA molecule. In hypoxia, HIF1α upregulates PDK1 expression, which, in turn, limits the amount of pyruvate molecules entering the citric acid cycle, leading to a decrease in oxygen consumption rate [48]. Inhibition of PDK1 results in an increase in the oxygen consumption rate in tumor cells and affects the glucose metabolism [40]. Therefore, this feature can be used to resensitize the tumor cells to radiotherapy. Also, targeting the M2 isoform of pyruvate kinase (PK) induces G2/M cell cycle arrest and promotes radiation-induced apoptosis and autophagy, thus increasing the radiosensitivity of non-small cell lung cancer (NSCLC) cells in vitro and in vivo [62].

Some key regulators and molecules involved in glucose metabolism (HIF1, GLUT1, and pyruvate kinase) can be used as prognostic and predictive markers in radiation therapy [60,63,64].

## 6. Radiotherapy Resistance and Mitochondrial Metabolism

Both glucose metabolism and mitochondrial biogenesis are related to radioresistance development. Mitochondria play a significant role in cancer cells through macromolecular synthesis and energy production. These organelles support rapid tumor growth by modulating energy metabolism [65]. The mitochondrial antioxidant manganese superoxide dismutase (MnSOD) is the main antioxidant enzyme that detoxifies superoxide radicals generated by mitochondrial respiration [66]. MnSOD protects cells from ROS-induced damage. In experiments with human pancreatic cancer cells, MnSOD overexpression significantly increased cell survival following irradiation with 6 Gy of gamma-radiation [67]. Similar results were obtained earlier in experiments, with MnSOD-overexpressing human ovarian cancer cells, human neuroblastoma tumor cells, hepatocellular carcinoma cells, and oral squamous carcinoma cells [42,68,69]. Activation of the G2 checkpoint has been demonstrated in eukaryotic cells in response to ionizing radiation [70]. It prevents cells from entering mitosis when DNA is damaged, thus providing an opportunity to maintain genomic stability. The regulation of cell cycle progression by ROS signaling may occur through the reduction or oxidation of critical cysteine in cell cycle regulatory proteins [44]. After exposure to ionizing radiation, there was a decrease in ATM, γH2AX, and cyclin B1 protein levels in MnSOD-overexpressing pancreatic cancer cells. Thus, MnSOD may regulate mitochondrial–nuclear interactions through the activation of the cell cycle checkpoint pathways. Demet Candas and colleagues reported that in HER2-positive breast cancer cells, irradiation leads to MKP1 (Mitogen-Activated Protein Kinase Phosphatase 1) translocation into mitochondria, where it prevents apoptotic induction by limiting the accumulation of phosphorylated active forms of the stress kinase JNK [71].

## 7. Radiotherapy Resistance and Cell Signaling Pathways

Upon exposure to ionizing radiation, tumor cells activate DNA damage repair, undergo the accelerated repopulation phase, and start to actively proliferate [72]. Various cellular mechanisms occurring during the repopulation phase lead to resistance to radiotherapy: DNA repair, cellular senescence, and cell cycle checkpoint regulation [73]. Several studies have shed light on the different genes related to innate and acquired radioresistance. Zhou and colleagues established a radioresistant breast cancer MDA-MB-231 subline and mouse xenografts to analyze the gene expression profiles; CDKN1A and MnSOD were found to be significantly upregulated in radioresistant cells [43]. High CDKN1A expression correlates with poor prognosis. CDKN1A is a cyclin-dependent kinase inhibitor, a major target of p53, and it regulates cell cycle progression at the G1 and S phases [74].

Cancer stem cells (CSCs) represent a distinct subpopulation within tumors, characterized by their capacity for self-renewal and DNA damage repair. These characteristics contribute to the resistance of CSCs to radiation therapy. They demonstrate low levels of ROS and exhibit slow rates of proliferation [75]. ROS scavengers (superoxide dismutase, superoxide reductase, catalase) are upregulated and exhibit high activity in CSCs across various tumors, resulting in reduced levels of ROS and providing protection to CSCs against radiation-induced cell death [76,77]. Also, the pro-survival pathways in CSCs appear to be more pronounced, protecting them from cell death. CSCs can evade treatment modalities through various intrinsic and extrinsic mechanisms. These mechanisms include resistance to oxidative DNA damage, upregulation of anti-apoptotic signaling pathways, and enhanced DNA repair [78]. In solid tumors, radiotherapy preferentially targets and eliminates bulk cancer cells population, thereby resulting in an enrichment of CSCs within the tumor [79].

Gene expression profiling has been used to identify radioresistance markers of the most common subtype of lung cancer—NSCLC; 18 genes have been shown to be associated with resistance, but only 3 of them were validated (MDM2, Livin α, TP54I3) [80]. The MDM2 gene encodes E3 ubiquitin–protein ligase, which represses p53 transcriptional activity [81]. MDM2 is associated with innate radiation resistance, and Livin α and TP54I3 are related to acquired radioresistance. In nasopharyngeal carcinoma, Livin α is an inhibitor of apoptosis. Significant differences in the efficacy of radiotherapy were observed between patients expressing Livin α and those who did not; among the 29 patients who did not express Livin α, the overall response rate to radiotherapy was 96.6% [82]. Also, Livin α was found to be downregulated in NSCLC cells after irradiation. On the other hand, the putative quinone oxidoreductase (TP54I3) is upregulated; it is induced by the p53 protein and is involved in apoptosis [80]. Y.S. Lee et al. detected a significant downregulation of tumor protein p53-inducible protein 3 (TP53I3) in a radioresistant NSCLC cell line [47].

Survivin is an inhibitor of apoptosis with a strong expression pattern in human cancers. It blocks the apoptotic pathway by inhibiting caspase 9 [83]. Survivin inhibits apoptosis and enhances cell viability, thus leading cancer cells to radioresistance [84]. The inhibition of Survivin causes a significant decrease in the cell viability of H460 lung cancer cells after irradiation. Similar results were observed in experiments with esophageal squamous cell carcinoma (ESCC) cells [85], cervical cancer cells [86], and oral squamous cell carcinoma cells [87]. Also, studies to date have shown that tumor radioresistance is associated with proliferation-regulating proteins; the Ras/Raf family members are constitutively activated and lead to radioresistance of laryngeal cancer [88]. A relationship between resistance and dysregulation of the phosphatidylinositol 3-Kinase AKT (PI3K/AKT) pathway has been reported in several human cancers, including head and neck [89], breast [90], colon [91], bladder [92], and fibrosarcoma [93].

Circulating non-invasive biomarkers might represent a significant tool for predicting radioresistance and radiosensitivity. Nasopharyngeal carcinoma (NPC) is a common head and neck malignancy; the standard treatment for NPC is radiotherapy. Unfortunately, radioresistance causes local recurrence and remains a serious obstacle to full recovery. Recently, serum microRNAs (miRNA) hsa-miR-1281 and hsa-miR-6732-3p have been reported as potential biomarkers for predicting the radiosensitivity of NPC [94]. Plasma exosomal miRNA miR-96 was found to be upregulated in patients with radioresistant NSCLC [95]. Moreover, miR-96 demonstrates a significant correlation with vascular invasion and poor overall survival. Also, extracellular miR-1246 has been reported to promote lung cancer cell proliferation and to enhance acquired radioresistance [96]. Its expression increased along with the irradiation dose received. MiR-1246 acts as a signaling messenger between different populations of cancer cells. It contributes to radioresistance by suppressing the key mediator of apoptosis DR5 [96]. C4b-binding protein alpha-chain (C4BPA) and vitronectin protein plasma levels might be used as indicators of radiation-induced lung toxicity. Both proteins belong to the inflammatory networks of TGF-beta-1 and interleukin-8; C4BPA and vitronectin play crucial roles in triggering radiation-induced damage in the lungs [97]. Also, radiotherapy is one of the main treatment options for cervical cancer and is used in more than 60% of cases [98]. Plasma miR-145 levels might be used for the diagnosis and radiosensitivity prediction of human cervical cancer [99]. MiR-145 downregulation was found to be associated with poor cancer differentiation, lymph node metastasis, and the advanced FIGO (The International Federation of Gynecology and Obstetrics) stage.

## 8. Conclusions

The response of specific malignant tumor cells to ionizing radiation depends on their radiosensitivity. Radiotherapy resistance develops in tumor cells due to either intrinsic or extrinsic factors and greatly reduces the efficiency of radiotherapy. The molecular mechanisms underlying the radioresistance are complicated. Several studies mentioned in this article demonstrate that radioresistance is associated with dysregulation of cancer cell metabolism, cell cycle control, apoptosis, and angiogenesis. These findings have partially contributed to the understanding of the mechanisms that underlie radioresistance. However, a substantial amount of work is yet to be completed before we clarify all molecular parameters that determine radiotherapeutic resistance and use this information to improve the therapy. In particular, we found only a few works dedicated to identifying circulating biomarkers of radioresistance and radiosensitivity in patients. We believe this is an unfortunate omission, since the acquired data can be an excellent groundwork for the development of radiotherapy sensitizers to overcome radioresistance in tumors. Additionally, biomarkers can be used to predict the resistance in patients in advance. At the same time, we should keep in mind that the radiotherapeutic resistance is not regulated by a single gene, but instead involves multiplex interactions of different cellular pathways.

## Figures and Tables

**Table 1:** Radiotherapy resistance biomarkers and their functions.

Biomarker	Cell Line	Expression Level	Function	Cell Line Model	Molecular Mechanism
**Breast cancer**					
Glucose transporter 1 (Glut1) [40,41]	MDA-MB-231, MCF-7	Upregulated	Glucose transport	Breast cancer, oral squamous cell carcinoma	Lactate is involved in radiation sensitivity
Cyclin-dependent kinase Inhibitor 1A (CDKN1A) [42]	MDA-MB-231	Upregulated	Cell cycle regulator	Breast cancer	CDKN1A regulates cell cycle progression at the G1 and S phases
**Lung cancer**					
Survivin [43]	NCI-H1299	Upregulated	Inhibition of apoptosis	Non-small-cell lung carcinoma	Survivin inhibits caspase 9 and blocks the apoptotic pathway
Pyruvate kinase M2 isoform [44]	A549	Upregulated	Glucose metabolism	Non-small-cell lung carcinoma	Contributes to radioresistance by generating a chemically reduced environment
Mouse double minute 2 homolog (MDM2) [45]	A549	Upregulated	Apoptosis regulator	Non-small-cell lung carcinoma	The level of MDM2 expression determines the extent to which radiation induces an increase in the activity of the TP53
Livin α [46]	A549	Upregulated	Inhibition of apoptosis	Non-small-cell lung carcinoma	Radiotherapy leads to Livin expression in tumor cells as a result of apoptosis tolerance, contributing to the radioresistance of lung cancer cells
Tumor protein p53 inducible protein 3 (TP53I)3 [47]	NCI-H460	Downregulated	Apoptosis regulator	Non-small-cell lung carcinoma	TP53I3 contributes to apoptosis, whereas the reduced expression is involved in acquired radioresistance
**Other cancers**					
Manganese superoxide dismutase (MnSOD) [48]	MIA PaCa-2	Upregulated	Antioxidant enzyme	Pancreatic cancer, ovarian cancer, neuroblastoma	Increased MnSOD activity alters the radiation-induced G2 checkpoint pathway
Pyruvate dehydrogenase kinase (PDK) [49]	U87	Upregulated	Glucose metabolism	Glioblastoma	PDK1 inhibits the citric acid cycle and activates glycolysis to promote stem-like traits
